# Development of 3-decen-2-one/HP-β-CD Inclusion Complex with Enhanced Stability and Its Application for Potato Postharvest Preservation

**DOI:** 10.3390/foods15142501

**Published:** 2026-07-15

**Authors:** Xia Ge, Yiwen Xu, Zheng Huang, Jiachun Tian, Mei Li, Jianxin Cheng, Shouqiang Li, Yumei Li, Yaqian Zhang

**Affiliations:** 1 Institute of Agricultural Products Storage and Processing, Gansu Academy of Agricultural Sciences, Lanzhou 730070, China; 18868007196@163.com (Y.X.); tianjiachunlz@126.com (J.T.); limei7877@126.com (M.L.); rangerkmc@163.com (J.C.); nkylsq@163.com (S.L.); axi_li@126.com (Y.L.); 13679463972@163.com (Y.Z.); 2 Animal Husbandry, Pasture and Green Agriculture Institute, Institute of Agricultural Quality Standards and Testing Technology, Gansu Academy of Agricultural Sciences, Lanzhou 730070, China; huangz4321@126.com

**Keywords:** 3-decen-2-one, hydroxypropyl-β-cyclodextrin, inclusion complex, characterization, potato postharvest preservation

## Abstract

3-Decen-2-one is a promising natural potato sprout suppressant, but its high volatility, poor stability and low water solubility severely restrict its practical application. To overcome these drawbacks, a 3-decen-2-one/hydroxypropyl-β-cyclodextrin (HP-β-CD) inclusion complex with a 1:1 host–guest molar ratio was successfully prepared via the co-evaporation method, and the stability constant was calculated to be 596.43 M^−1^ by phase solubility studies. Molecular modeling demonstrated that the inclusion complex was primarily stabilized by van der Waals interactions, and the optimal binding conformation was that the alkyl chain of 3-decen-2-one inserted into the cavity from the wider rim of HP-β-CD. The inclusion complex was further characterized by ^1^H NMR, FT-IR, TG, and SEM. 3-Decen-2-one showed a better water solubility, thermal stability and release properties after complexation. In the potato storage evaluation, the complex exhibited greater sprout inhibition than free 3-decen-2-one; effectively reduced weight loss, the respiration rate, and MDA accumulation; and maintained a higher Vc and lower reducing sugar levels. Therefore, the obtained inclusion complex is a promising approach to stabilize and achieve the sustained release of 3-decen-2-one as a natural sprout inhibitor for potato storage.

## 1. Introduction

Potatoes (*Solanum tuberosum* L.) are the fourth most important food crop worldwide, and their high-yield potential and rich nutritional value have made them a widely cultivated cash crop in many countries [1]. In terms of the global food crisis, potatoes play an important role, especially in solving food shortages, a lack of nutrition, and excessive price increases [2,3]. However, the post-dormancy sprouting of potato tubers results in compromised nutritional and processing quality, characterized by tuber shrinkage, increased weight loss, accelerated decay, and the accumulation of toxic glycoalkaloids (e.g., solanine and chaconine) [4,5,6]. It is a significant cause of quality degradation and economic losses in the potato industry. For decades, chlorpropham (CIPC) has been the primary chemical sprout inhibitor due to its low dosage and high efficacy. Nevertheless, in recent years, growing regulatory concerns regarding its residues have created an urgent need to explore alternative methods for inhibiting potato sprouting during storage [6].

3-Decen-2-one is a volatile α,β-unsaturated aliphatic ketone naturally occurring in various sources such as mushrooms [7]. It has been approved as a food additive and flavoring agent in many regions [8]. This compound has gained increasing attention as a potential potato sprout inhibitor due to its remarkable efficacy and favorable safety profile [9], and it has obtained valid biopesticide registrations for potato sprout inhibition in Canada and the United States [10,11]. Typically administered as a thermal fog or aerosol in enclosed storage facilities at the stage of potato dormancy break, 3-decen-2-one exerts its sprout-inhibiting effect through vapor–tuber interactions [12]. However, similar to other volatile active substances, 3-decen-2-one faces several challenges that limit its practical application as a novel sprout inhibitor. Its low water solubility, high volatility, and poor thermal stability lead to a low bioavailability and require frequent reapplication in practice. Moreover, the direct dropping of the raw chemical on tubers can induce peel burning and deteriorate the sensory appearance [2,7].

Cyclodextrins are cyclic oligosaccharides able to encapsulate hydrophobic molecules within their hydrophobic cavities, forming molecular-level inclusion complexes [13] and thereby enhancing the physical, chemical, and biological properties of the encapsulated guest molecules [14]. Especially for natural plant essential oils and volatile compounds, CDs can improve their properties such as solubility, stability, and volatility [15]. Santos et al. [16] prepared a β-citronellol/β-cyclodextrin (β-CD) inclusion complex to improve the solubility and bioavailability of β-citronellol, and markedly enhanced its anti-hyperalgesic activity. Guan et al. [17] reported that hydroxypropyl-β-cyclodextrin (HP-β-CD) encapsulation effectively improved the photostability, thermal stability and volatility resistance of star anise alcohol extracts. Huang et al. [18] encapsulated CIPC with hydroxypropyl-β-cyclodextrin to improve CIPC’s water solubility, dissolution rate and thermal stability, and the inclusion complex achieved equivalent sprout-inhibiting effects with lower dosages of the chemical sprout inhibitor CIPC. Nevertheless, these studies only focused on improving the stability and bioavailability of guest molecules; the humidity-responsive controlled-release characteristics have not been investigated. A high relative humidity was widely adopted during potato storage to reduce weight loss and corresponding economic losses [19]. Meanwhile, such humid conditions can weaken intermolecular interactions between cyclodextrins and guest molecules and thus induce the sustained slow release of encapsulated active components [20].

Thus, in this study, HP-β-CD was selected to form an inclusion complex with 3-decen-2-one, which is a hydroxyalkyl β-CD derivative with a better inclusion ability, a higher water solubility and a lower toxicity [21]. The prepared complexes were characterized and subsequently applied to potato storage. This approach was intended to enhance the stability of 3-decen-2-one and achieve its controlled release. This study provides theoretical and practical insights for the application of encapsulated volatile compounds in potato sprout inhibition and preservation.

## 2. Materials and Methods

### 2.1. Materials

HP-β-CD (0.6 molar substitution, average Mw ~ 1380) was purchased from Sigma-Aldrich (Shanghai, China). 3-Decen-2-one (purity > 93%) was obtained from Macklin Biochemical Co., Ltd. (Shanghai, China). Potatoes of the “Longshu 7” cultivar were obtained from Gansu Yihang Potato Industry Technology Development Co., Ltd. (Lanzhou, China). All other reagents were of analytical grade.

### 2.2. Phase Solubility

The stability constant was determined via the phase solubility method as reported by Higuchi and Connors [22]. An excess amount of 3-decen-2-one (0.1 mL) was added to 10 mL colorimetric tubes containing HP-β-CD solutions of different concentrations (0, 10, 20, 30, 40, 50 mM). The mixtures were thoroughly shaken at 20 °C, and upon reaching equilibrium, the aqueous phase was filtered through a 0.22 μm membrane filter and appropriately diluted. The content of 3-decen-2-one in the solution was determined using a UV-1900 UV–visible spectrophotometer (Shimadzu Corporation, Kyoto, Japan), with the absorbance measured at 230 nm. All experiments were performed in triplicate.

According to the Higuchi–Connors equation,

(1) $$K=\frac{slope}{S_{0}(1-slope)}$$

where *K* is the stability constant of the inclusion complex at 20 °C and *S*_0_ is the solubility of 3-decen-2-one in water without cyclodextrin.

Furthermore, the *K* of the complex at 25 °C, 30 °C, 35 °C and 40 °C was also determined to obtain the relevant thermodynamic parameters of the inclusion process. The enthalpy change (Δ*H*) and entropy change (Δ*S*) of the inclusion complexation were calculated using the Van’t Hoff equation (Equation (2)), and the Gibbs free energy (Δ*G*) was determined by Equation (3).

(2) $$lnK=-\frac{\Delta H}{RT}+\frac{\Delta S}{R}$$

(3) ΔG=ΔH−TΔS

where *R* is the gas constant (8.314 J mol^−1^ K^−1^) and *T* represents the absolute temperature (K). The obtained data were used to analyze the thermodynamic behavior of the inclusion process.

### 2.3. Preparation of 3-decen-2-one/HP-β-CD Inclusion Complex and Their Physical Mixture

The 3-decen-2-one/HP-β-CD inclusion complex was prepared by the co-evaporation method in a three-neck round-bottom flask equipped with a reflux device. Specifically, 4.0 mmol of HP-β-CD was dispersed in 24 mL of distilled water and magnetically stirred until fully dissolved. A 6 mL ethanolic solution containing 4.0 mmol of 3-decen-2-one was then added dropwise into the above HP-β-CD aqueous solution under continuous stirring. The mixture was heated at 80 °C with magnetic stirring for 4 h, followed by stirring at room temperature for an additional 2 h. After ethanol was evaporated by a rotary evaporator, the solution was filtered and lyophilized. The obtained complex was sealed and stored in a dry and low-temperature environment. Under the above preparation conditions, the maximum yield of the complex reached 87.99%. After the measurement, in the complex, the content of 3-decen-2-one was 9.82 wt.%.

In addition, HP-β-CD and 3-decen-2-one were accurately weighed according to 1:1 (molar ratio) and quickly mixed thoroughly to obtain the physical mixture prior to the related characterization experiments.

### 2.4. Characterization of the 3-decen-2-one/HP-β-CD Complex

Molecular docking was performed using the AutoDock Vina 1.1.2 software. The structures of the guest molecule 3-decen-2-one and the host receptor HP-β-CD were made as follows: their SDF files were downloaded from PubChem, converted to the PDB format using PyMOL 2.3, and then loaded into AutoDockTools 1.5.7 to assign atomic types and add atomic charges, and were finally saved in the PDBQT format. Commercial HP-β-CD exists as a mixture of hydroxypropyl-substituted isomers. A standard strategy widely adopted in cyclodextrin simulation studies is to select a representative conformation for modeling [23]. According to our experimental molecular weight data, the HP-β-CD sample exhibited a characteristic substitution profile where three glucose residues are functionalized with hydroxypropyl groups. Therefore, we retrieved the SDF file of HP-β-CD (PubChem CID 14049689, computed by PubChem 2.2) and randomly retained three hydroxypropyl substituents on its backbone. This representative structure relatively accurately reflects the characteristics of the major components in practical HP-β-CD samples. The guest molecule was set as flexible, and HP-β-CD was defined as rigid merely for rapid preliminary pose screening. The exhaustiveness of the search was set to 100, with other parameters at the default. The coordinates of the docking box center were: center_x = 14.5, center_y = 5.6, center_z = 38.16; the dimensions of the docking box were: size_x = 20, size_y = 20, size_z = 20. The optimal docking conformation was selected as the initial structure for subsequent molecular dynamics simulations.

Molecular dynamics (MD) simulations were carried out with the GROMACS 2025 software package. Both molecules adopted the GAFF2 force field, and the system was solvated with the TIP3P water model. Periodic boundary conditions and the particle-mesh Ewald (PME) method were applied, with a 14 Å cutoff for short-range interactions. The system underwent 5000 steps of steepest-descent energy minimization, followed by 100 ps position-restrained MD in NVT and NPT ensembles. Unrestrained production simulations were run for a minimum of 200 ns in the NPT ensemble at 298.15 K and 1 atm, controlled by velocity rescaling and Berendsen algorithms, respectively. A 2.0 fs time step and LINCS bond constraints were applied throughout. Trajectory analyses, including the root mean square deviation (RMSD), radius of gyration (Rg), solvent-accessible surface area (SASA), hydrogen bond distribution, and energy evolution, were conducted to evaluate the system convergence and structural stability.

^1^H NMR spectra of HP-β-CD and the 3-decen-2-one/HP-β-CD inclusion complex were recorded individually on a Bruker AVANCE(III) HD 600 spectrometer (Bruker Instruments Inc., Billerica, MA, USA). Deuterium oxide (D_2_O, 99.9 atom% D, containing 0.03% (*v*/*v*) tetramethylsilane) was used as the NMR solvent, with the residual water peak at 4.64 ppm being used as the internal standard. The acquisition parameters were set as follows: spectral width = 4000 Hz, acquisition time = 3.98 s, number of scans = 8, and relaxation delay = 1 s.

FT-IR spectra of 3-decen-2-one, HP-β-CD, their physical mixture and the complex samples were recorded by a Fourier-transform infrared spectroscopy (Thermo Nicolet Avatar 360, Thermo Fisher Scientific, Madison, WI, USA), ranging from 4000 to 400 cm^−1^ by using KBr pellets.

A thermogravimetry (TG) analysis was performed using an STA449F5 thermal analyzer (Netzsch, Selb, Germany). Approximately 1–2 mg samples of 3-decen-2-one, HP-β-CD, their physical mixture, and the complex were accurately weighed and placed in sealed alumina crucibles for the measurements, respectively. The heating rate was set at 10 °C·min^−1^ within the temperature range from 25 to 500 °C in nitrogen.

Scanning electron microscopy (SEM) images were acquired on a JSM-7800F ultra-high-resolution thermal field-emission scanning electron microscope (JEOL Ltd., Tokyo, Japan). The samples were mounted on sample stubs, sputter-coated with platinum, and subsequently observed at an accelerating voltage of 20.0 kV with magnifications of 2000× and 4000×, respectively.

### 2.5. Release Characteristic of 3-decen-2-one from Different Matrices Under Different Environmental Conditions

According to the storage environments for 3-decen-2-one and potato tubers described by Pringle et al. [19], two environmental conditions (15 °C, 40% RH and 15 °C, 95% RH) were selected to perform 3-decen-2-one release studies, which were precisely controlled using a constant temperature and humidity chamber (KMH-64, KOMEG Technology Ind Co., Ltd., Dongguan, China). The release behavior of 3-decen-2-one from three different matrices was investigated: (1) filter paper, where 0.1 g 3-decen-2-one was accurately weighed and uniformly spread onto the filter paper; (2) a diatomite mixture, containing 10 wt.% 3-decen-2-one; and (3) an inclusion complex, with the drug-loading quantity of 9.82 wt.%. A total of 1 g of each solid sample was accurately weighed. The samples were taken at 24 h intervals for 7 days. The residual 3-decen-2-one retained on filter paper was measured gravimetrically, while the residual amounts in powder matrices were quantified by UV spectrophotometry. The retention rate of 3-decen-2-one in different matrices was expressed as:

(4) $$R\left(\%\right)=\frac{m}{m_{0}}\times 100$$

where *R* is the retention rate, *m* is the residual amounts of 3-decen-2-one in different matrices for a certain time, and *m*_0_ is the initial 3-decen-2-one content in different matrices.

### 2.6. Potato Tuber Storage Experiments

#### 2.6.1. Treatments

Fresh potatoes with a uniform size, intact skin, and an individual weight of 150–200 g were selected as experimental materials. Nine corrugated cartons (55 cm × 40 cm × 30 cm) were prepared, and approximately 20 kg of potato tubers were loaded into each carton, occupying 2/3 of the total internal volume. The tubers were subjected to wound healing at 15 °C and 95% RH, and subsequent storage under the same conditions [24]. Potato dormancy was broken after 2–3 months postharvest, and the treatments were implemented uniformly at the sprout-peeping stage. Three experimental treatments were conducted as follows:

(1) Control group: No sprout-suppressing agents were applied.

(2) Free 3-decen-2-one treatment: The application dosage was set at 0.12 mL kg^−1^ based on commercial recommended usage [2]. A total of 240 μL of pure 3-decen-2-one was evenly dropped onto a 2.0 cm × 2.0 cm filter paper and sealed inside a 2.5 cm × 5.0 cm non-woven fabric sachet. Ten identical sachets were evenly distributed among potato tubers per carton.

(3) 3-Decen-2-one/HP-β-CD inclusion complex treatment: The amount of the complex was calculated to provide an equimolar amount of 3-decen-2-one equal to that applied in Treatment 2. Specifically, 2.0 g of inclusion complex powder was sealed into a non-woven fabric sachet of the same size as above, and ten such sachets were scattered throughout a potato tuber pile.

Three independent biological replicates were arranged separately for each treatment. During storage, each carton was opened for 10 min of natural ventilation weekly. Observations were taken at 14-day intervals throughout the experiment for 70 days. The sprout length, sprouting rate, weight loss, respiration rate, malondialdehyde (MDA), vitamin C (Vc), and reducing sugar contents were measured. For weight loss measurement, 10 fixed tubers from each replicate were repeatedly weighed throughout storage, and other physiological indicators were determined using randomly sampled tubers.

#### 2.6.2. Determination Methods of Indicators

Sprout length was measured using a vernier caliper and expressed in millimeters (mm). Tubers were judged as sprouting when the length of the longest sprout exceeded 2 mm [25]. The sprouting rate (%) was calculated as the percentage of the number of sprouting tubers compared to the total number of tubers.

Weight loss was determined following the method described by Zhang et al. [26], calculated based on the variation in tuber mass at different storage times. The results were expressed as a percentage (%).

The respiration rate was measured using a CA-10 Carbon Dioxide Analyzer (Sable Systems International, North Las Vegas, NV, USA) and expressed as carbon dioxide (CO_2_) production in mg CO_2_ kg^−1^ h^−1^ [27].

The MDA content was determined using the thiobarbituric acid (TBA) method described by Quandahor et al. [28].

The Vc content in potatoes was measured by the 2,6-dichloroindophenol (DCIP) titration method [29].

The reducing sugar content was determined following the method of Zhang and Lu [30].

### 2.7. Statistical Analysis

All data were organized using Microsoft Excel 2021 (Microsoft Corporation, Seattle, WA, USA). The statistical analysis was performed using the SPSS Statistics 19.0 software (SPSS Inc., Chicago, IL, USA). A one-way analysis of variance (ANOVA) was adopted for data comparison, and Duncan’s multiple range test was used for post hoc multiple comparisons at the significance level of *p* < 0.05. All experiments were carried out in triplicate.

## 3. Results and Discussion

### 3.1. Phase-Solubility Analysis

**Figure 1:**
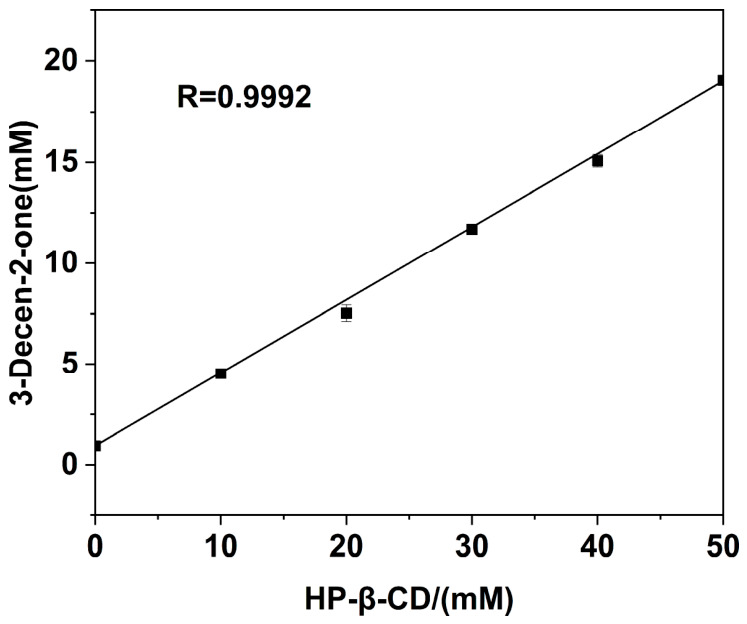
Phase solubility diagram of 3-decen-2-one in the presence of HP-β-CD.

The phase solubility study enabled the determination of the apparent stability constant of the inclusion complex [31]. Figure 1 shows the phase solubility diagram of 3-decen-2-one in HP-β-CD solutions with different concentrations. The solubility of 3-decen-2-one presented a linear increase with a rising HP-β-CD concentration. According to the Higuchi–Connors theory, the phase solubility diagram is classified as the A_L_ type, indicating the formation of a water-soluble inclusion complex with a 1:1 host–guest molar ratio [32,33]. The stability constant *K* of the complex was calculated to be 596.43 M^−1^ using Equation (1). When the concentration of HP-β-CD in the solution reached 50 mM, the water solubility of 3-decen-2-one was increased by 20.10 times. These results demonstrated that the water solubility of 3-decen-2-one was significantly enhanced after the formation of the inclusion complex with HP-β-CD.

**Figure 2:**
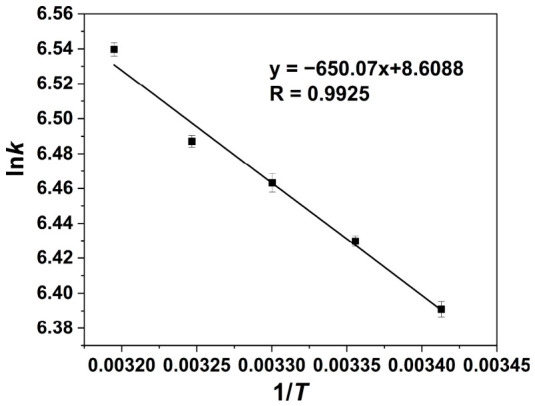
Van’t Hoff plot of ln *K* versus 1/*T*.

Van’t Hoff plot based on Equation (2) was used to calculate the thermodynamic parameters for the 3-decen-2-one/HP-β-CD complexation (Figure 2). As depicted in Table 1, the Δ*H* value was 5.40 KJ mol^−1^, indicating that inclusion complex formation was an endothermic process [34,35]. All negative Δ*G* values confirmed spontaneous inclusion, while positive Δ*S* (71.57 J mol^−1^ K^−1^) indicated increased disorder of the host–guest system. Thus, the inclusion was entropy-driven, with a hydrophobic effect as the dominant macroscopic driving force. The expulsion of ordered cavity water provided a large entropy gain, offsetting the desolvation energy cost of the host and guest and enabling spontaneous binding.

### 3.2. Characterization of the Inclusion Complex

#### 3.2.1. Molecular Modeling Studies

Molecular modeling studies provide insights into the binding modes and stability of cyclodextrin–small molecule inclusion complexes at the molecular level [36,37]. A total of nine clusters of docking conformations were generated from molecular docking, and a visualization analysis revealed that the small molecule tended to enter the wider rim of the HP-β-CD cavity via its ketone carbonyl end, which corresponded to a low-energy binding state. The conformation with the lowest binding affinity (−3.60 kcal mol^−1^) was selected as the initial structure for subsequent MD simulations.

**Figure 3:**
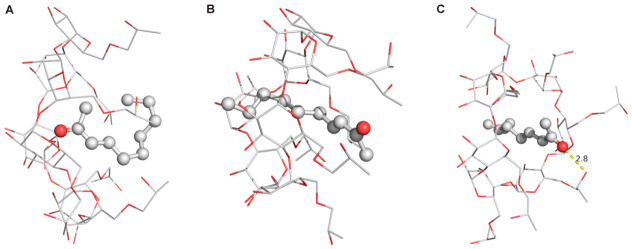
Dynamic conformational changes of the HP-β-CD/3-decen-2-one complex during molecular dynamics simulation: (**A**) initial docking conformation at 0 ns, where the carbonyl group of 3-decen-2-one inserted into the HP-β-CD cavity and the alkyl tail was exposed to the aqueous environment; (**B**) intermediate equilibrium conformation at 100 ns after the mutual induced fit, in which the alkyl tail was gradually buried into the hydrophobic cavity and the carbonyl group turned toward the outer rim of HP-β-CD; and (**C**) stable equilibrium conformation at 200 ns, where the carbonyl oxygen was exposed to the solvent and formed an intermolecular hydrogen bond (length = 2.8 Å) with the hydroxyl group of HP-β-CD.

The host–guest binding geometries at 0 ns (initial docking conformation), 100 ns and 200 ns of the molecular dynamics simulations were shown in Figure 3. During the 200 ns simulation, obvious conformational rearrangement was observed. Different from the initial docking pose (0 ns), where the carbonyl group of 3-decen-2-one inserted into the HP-β-CD cavity, the complex gradually reached a thermodynamically stable state after a mutual induced fit. The hydrophobic alkyl chain of 3-decen-2-one fully embedded inside the HP-β-CD cavity, while the carbonyl group was exposed to the external aqueous environment. This stable conformation remained unchanged from 100 ns to 200 ns, indicating that the optimal docking pose was not the stable binding configuration. In addition, the carbonyl group of the guest molecule formed hydrogen bonds with the hydroxypropyl substituents of HP-β-CD.

**Figure 4:**
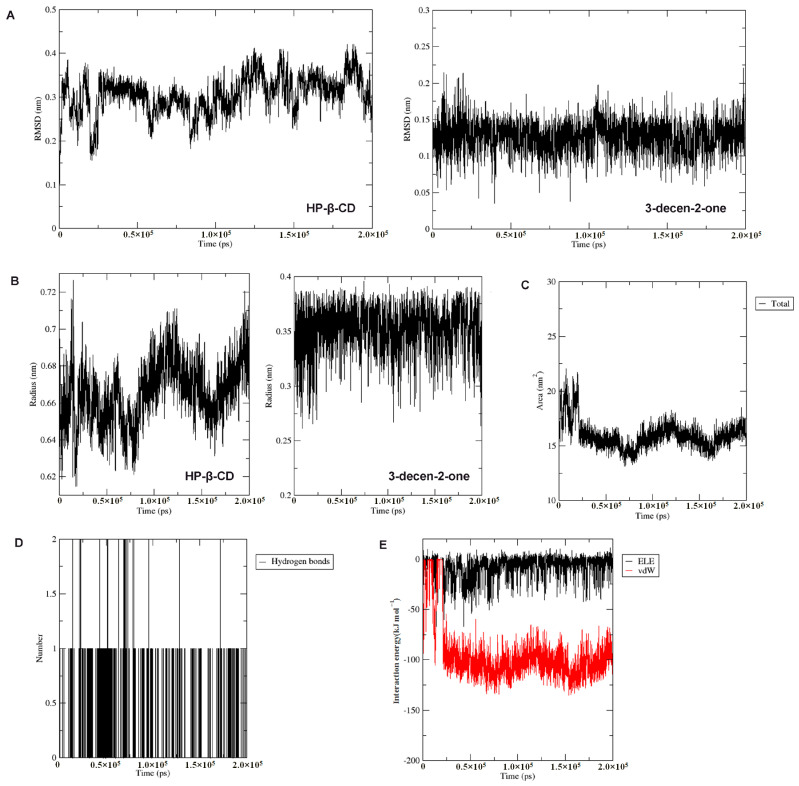
Trajectory analysis of the HP-β-CD/3-decen-2-one complex over the 200 ns MD simulation. (**A**) RMSD curves of HP-β-CD (**left**) and 3-decen-2-one (**right**); (**B**) Rg profiles of HP-β-CD (**left**) and 3-decen-2-one (**right**); (**C**) total SASA of the complex; (**D**) number of intermolecular hydrogen bonds formed between the host and guest; and (**E**) MM-GBSA decomposition energy: van der Waals energy (black) and electrostatic energy (red).

The RMSD and Rg curves were analyzed to evaluate the structural stability of the complex. As shown in the RMSD and Rg curves (Figure 4A,B), HP-β-CD presented a slight conformational fluctuation due to its high hydrophilicity and strong interaction with surrounding water molecules. In contrast, the encapsulated 3-decen-2-one maintained steady RMSD (~0.15 nm) and Rg (~0.35 nm) values throughout the trajectory, demonstrating that the guest was tightly confined within the cyclodextrin cavity. The total SASA of the complex was further monitored over the 200 ns trajectory (Figure 4C). The high initial SASA at 0 ns arose from the fully exposed alkyl chain of 3-decen-2-one in the docking pose. During the mutual induced fit, the alkyl chain inserted into the HP-β-CD cavity, with only the polar carbonyl group exposed to bulk water, sharply reducing the overall SASA within 20 ns. The SASA fluctuated mildly at 14–18 nm^2^ from 20 to 200 ns without drift, jointly verifying structural equilibrium together with stable RMSD and Rg curves. The lowered-equilibrium SASA highlighted hydrophobic encapsulation as the main binding driving force. A hydrogen bond analysis (Figure 4D) revealed that HP-β-CD and 3-decen-2-one formed an average of one hydrogen bond throughout the entire MD simulation, which further stabilized the inclusion structure.

MM-GBSA calculations were carried out using 250 equidistant snapshots extracted from the 50–100 ns trajectory (Table 2), and the total binding free energy (ΔG_total_) of the complex was −22.03 kcal mol^−1^. The binding process was mainly driven by van der Waals forces (−25.64 kcal mol^−1^), followed by electrostatic interactions (−2.11 kcal mol^−1^) and nonpolar solvation effects (−3.57 kcal mol^−1^). Consistent with the free energy results, the dynamic interaction energy curves (Figure 4E) proved that the van der Waals force (~−100 kJ mol^−1^) was markedly stronger than the electrostatic force (−10 kJ mol^−1^), which confirmed that the van der Waals interaction was the predominant intermolecular force responsible for the stable encapsulation of 3-decen-2-one by HP-β-CD.

#### 3.2.2. ^1^H NMR Analysis

As shown in Table 3, the Δ*δ* values of H-3 and H-5, which are located within the hydrophobic cavity of HP-β-CD [38], are only +0.003 ppm and +0.011 ppm, respectively. Generally, after complexation, the H-3 and H-5 protons will exhibit obvious chemical shift changes owing to the anisotropic magnetic effect induced by the unsaturated groups of the guest molecule [39]. According to the molecular modeling results, the stable inclusion mode of the complex, the alkyl chain of 3-decen-2-one, might enter into the cavity from the wider rim of HP-β-CD, whereas its carbonyl group was exposed to the external aqueous environment. Accordingly, the carbonyl group of 3-decen-2-one did not enter into the HP-β-CD cavity and scarcely altered the microenvironment of the inner H-3 and H-5 protons.

#### 3.2.3. FT-IR Analysis

**Figure 5:**
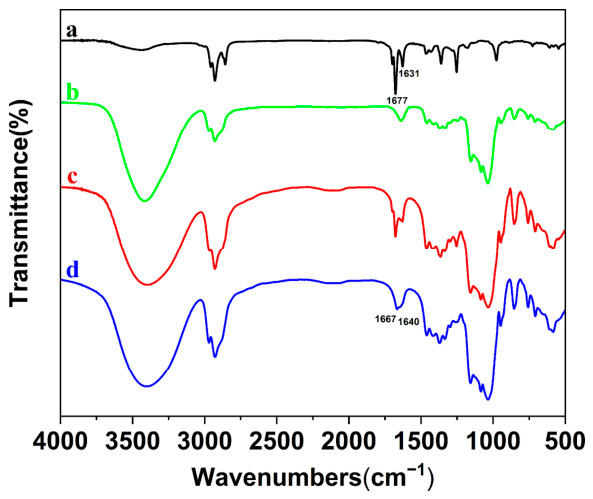
FT-IR spectra of 3-decen-2-one (a), HP-β-CD (b), physical mixture (c) and 3-decen-2-one/HP-β-CD inclusion complex (d).

The FT-IR spectra of 3-decen-2-one, HP-β-CD, their physical mixture, and the 3-decen-2-one/HP-β-CD inclusion complex are depicted in Figure 5. The characteristic peaks of 3-decen-2-one (Figure 5a) were assigned to the C=O stretching vibration (1677 cm^−1^) and C=C stretching vibration (1631 cm^−1^), which are typical absorption bands of α,β-unsaturated ketones. As shown in Figure 5c, the FT-IR spectrum simply superimposed the original characteristic peaks of HP-β-CD (Figure 5b) and 3-decen-2-one, indicating no intermolecular interaction between the host and guest in the physical mixture. In contrast, the spectrum of the inclusion complex (Figure 5d) exhibited obvious changes after encapsulation. The C=O stretching vibration of 3-decen-2-one shifted from 1677 cm^−1^ to 1667 cm^−1^, and the C=C stretching vibration shifted from 1631 cm^−1^ to 1640 cm^−1^. According to molecular modeling results, the unsaturated C=C–C=O moiety of 3-decen-2-one formed intermolecular hydrogen bonds with the hydroxypropyl groups of HP-β-CD. Hydrogen bonding weakened the C=O bond and induced its red shift, while the reduced π-conjugation between C=C and C=O resulted in a blue shift of the C=C stretching peak.

#### 3.2.4. Thermoanalysis

**Figure 6:**
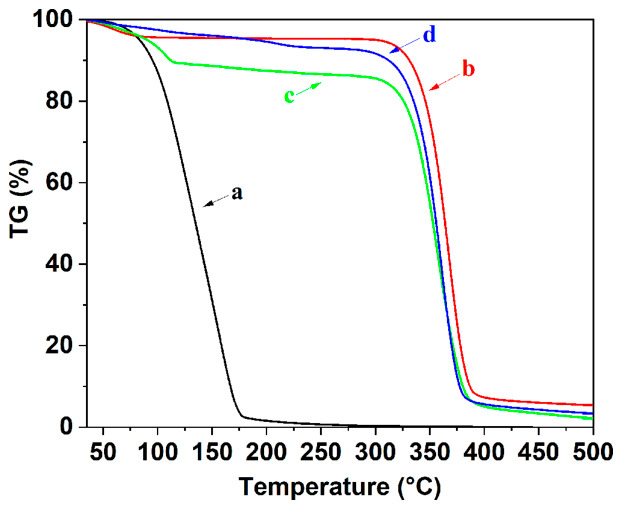
TG curves of 3-decen-2-one (a), HP-β-CD (b), physical mixture (c) and 3-decen-2-one/HP-β-CD inclusion complex (d).

A thermoanalysis is a commonly used technique for characterizing inclusion complexes that can verify the formation of host–guest inclusion complexes and evaluate the thermal stability changes of guest molecules before and after complexation [40]. Figure 6 presents the TG curves of 3-decen-2-one (a), HP-β-CD (b), their physical mixture (c) and the inclusion complex (d). As shown in Figure 6a, 3-decen-2-one exhibited an immediate weight loss starting at room temperature, followed by a sharp, near-complete mass loss in the range of 75 °C to 175 °C. This pronounced thermal behavior confirmed the high volatility and low thermal stability of free 3-decen-2-one. HP-β-CD presented a slight weight loss occurring between 50 °C and 100 °C, corresponding to the evaporation of physically adsorbed moisture, while major thermal decomposition takes place in the 300–325 °C range (Figure 6b). For the physical mixture (Figure 6c), gradual weight loss from 35 °C to 125 °C resulted from moisture evaporation and 3-decen-2-one volatilization. Between 300 °C and 325 °C, its thermal weight loss behavior was similar to that of pure HP-β-CD, indicating that the thermal characteristic of cyclodextrin dominated and no strong host–guest interactions existed in the physical mixture. In contrast, the inclusion complex (Figure 6d) exhibited a steady, slow weight loss across the 25–300 °C range, with no abrupt mass loss events characteristic of free 3-decen-2-one. Furthermore, the temperature at which significant weight loss begins was substantially delayed compared to the physical mixture. This finding was consistent with previous reports [41,42]. These results confirmed that 3-decen-2-one was successfully encapsulated by HP-β-CD.

#### 3.2.5. SEM Analysis

**Figure 7:**
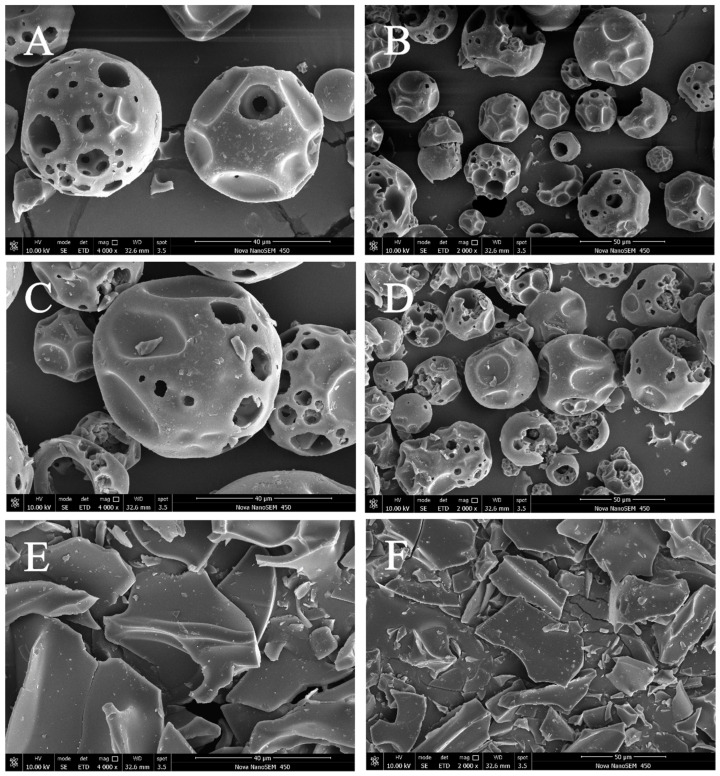
SEM images of HP-β-CD (**A**,**B**), physical mixture (**C**,**D**), and 3-decen-2-one/HP-β-CD inclusion complex (**E**,**F**). A, C, E: magnified 4000×; (**B**,**D**,**F**): magnified 2000×.

The surface morphology of HP-β-CD (Figure 7A,B), the physical mixture (Figure 7C,D) and the 3-decen-2-one/HP-β-CD inclusion complex (Figure 7E,F) was observed by scanning electron microscopy. HP-β-CD had spherical shapes with hollow centers. The physical mixture only showed physical adhesion between the two components, with no evident host–guest interactions. In contrast, the inclusion complex exhibited an irregular flake or plate-like morphology and a completely different phase structure. Similar morphological changes have been reported in essential oil/HP-β-CD and flavonoid/HP-β-CD inclusion complexes [43,44].

### 3.3. Release Characteristic of 3-decen-2-one from Different Matrices

**Figure 8:**
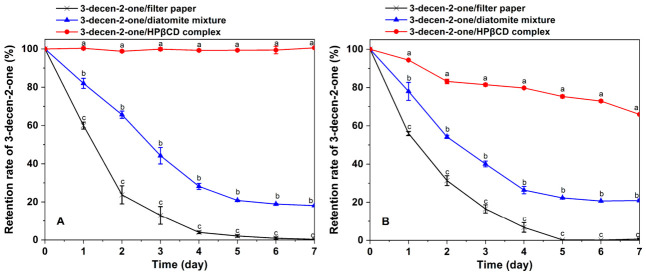
Retention rate of 3-decen-2-one from different matrices under different environmental conditions: (**A**) 15 °C, 40% RH; (**B**) 15 °C, 95% RH. Each value is the mean of three replicates. The vertical bars represent the standard deviation of the means. The different letters indicate significant differences (one-way ANOVA, *p* < 0.05).

As shown in Figure 8, the retention rates of 3-decen-2-one on filter paper and in the diatomite mixture were significantly lower than those in the inclusion complex (*p* < 0.05). At 15 °C and 40% RH (Figure 8A), the retention rates of the filter paper and diatomite mixture groups declined to 0.33% and 18.10% by Day 7, respectively. At 15 °C and 95% RH (Figure 8B), almost no residual 3-decen-2-one was detected in the filter paper group after Day 5, and the retention rate of the diatomite mixture group stabilized at approximately 21% from Day 5 to Day 7. In contrast, the complex maintained more than 98% of the initial 3-decen-2-one throughout the whole 7 days under dry conditions (15 °C, 40% RH), and approximately 65.94% of 3-decen-2-one still remained at Day 7 under high humidity (15 °C, 95% RH).

The release profiles of 3-decen-2-one from filter paper and diatomite were barely affected by the relative humidity. However, ambient humidity exerted a prominent effect on the release behavior of the inclusion complex. The complex exhibited an excellent stability under low-humidity conditions, whereas a high relative humidity significantly accelerated the escape of encapsulated 3-decen-2-one from the complex. This humidity-responsive release characteristic could be explained by competitive water displacement within cyclodextrin cavities [45]. These findings demonstrated that the 3-decen-2-one/HP-β-CD inclusion complex presented a superior sustained-release performance.

### 3.4. Evaluation of 3-decen-2-one/HP-β-CD Complex on Potato Preservation

#### 3.4.1. Sprout Length and Sprouting Rate

**Figure 9:**
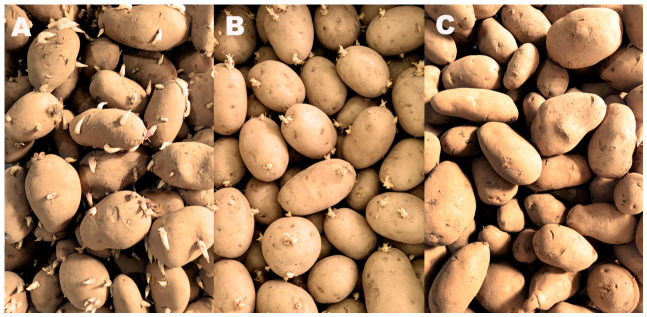
Potato sprout inhibition of different treatments after 70 d storage at 15 °C, 95% RH. (**A**) control; (**B**) 3-decen-2-one; and (**C**) 3-decen-2-one/HP-β-CD complex.

**Figure 10:**
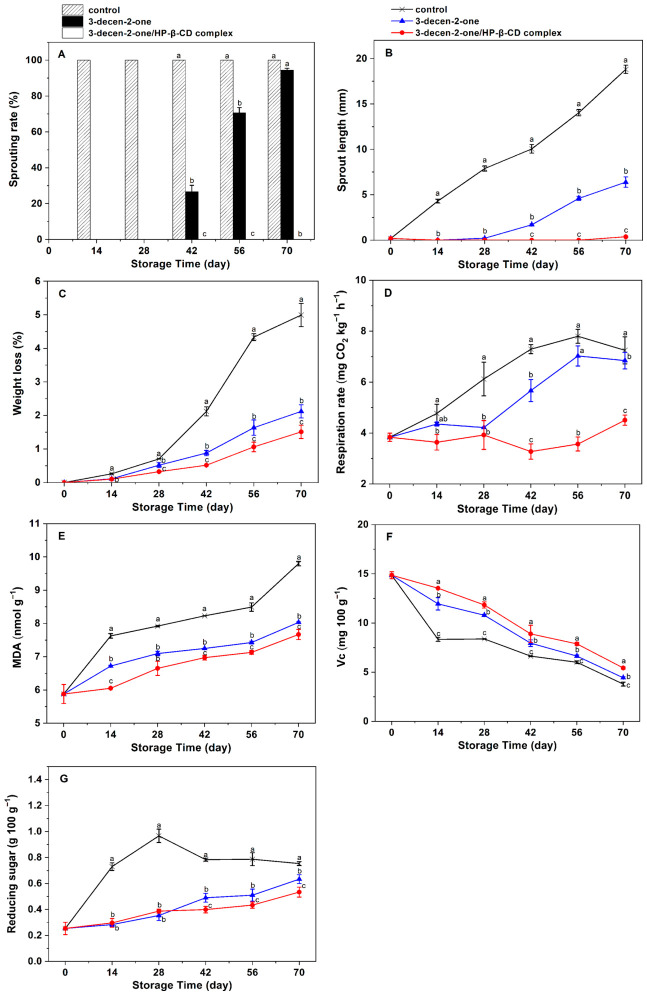
Effects of different treatments (control, 3-decen-2-one, 3-decen-2-one/HP-β-CD complex) on the sprouting rate (**A**), sprout length (**B**), weight loss (**C**), respiration rate (**D**), MDA content (**E**), Vc content (**F**) and reducing sugar content (**G**) of potatoes during storage. Each value is the mean of three replicates. The vertical bars represent the standard deviation of the means. The different letters indicate significant differences (one-way ANOVA, *p* < 0.05).

Figure 9 shows the potato tuber sprouting status of each group on the 70th day of storage. On the 70th day after treatment, the sprouting rate and sprout length of the potatoes in the control group were 100% and 18.82 mm, respectively, while those in the 3-decen-2-one treatment group were 94.44% and 6.39 mm. Meanwhile, both the sprouting rate and sprout length of the group treated with the 3-decen-2-one/HP-β-CD inclusion complex were significantly lower than those of the other treatments (*p* < 0.05). Almost no sprouting was observed throughout the entire storage period, and the sprout length was only 0.37 mm (Figure 10A,B). Notably, the 3-decen-2-one treatment exerted an excellent inhibitory effect on potato sprouting during the early storage stage (0–28 d). However, with the extension of storage time, the inhibitory effect gradually decreased due to the high volatility and rapid loss of 3-decen-2-one, resulting in obvious sprouting and sprout elongation. This finding was consistent with previous reports indicating that 3-decen-2-one requires multiple applications to achieve effective sprout inhibition at higher temperatures [2]. Compared with other treatments, the 3-decen-2-one/HP-β-CD complex treatment maintained effective sprout inhibition even after 70 days of storage. It was speculated that the inclusion complex may slowly and continuously release 3-decen-2-one during potato storage, which could potentially prolong the effective period of sprout control.

#### 3.4.2. Weight Loss

Potato sprouting is a key factor leading to the rapid increase in weight loss during storage. As shown in Figure 10C, potatoes in the control group exhibited the fastest water loss due to vigorous sprouting, reaching 4.99% on the 70th day of storage. The weight loss of potatoes treated with 3-decen-2-one was 2.12%, while the 3-decen-2-one/HP-β-CD inclusion complex treatment exhibited the lowest weight loss of only 1.51%, significantly lower than the other two treatments (*p* < 0.05). The result demonstrated that the 3-decen-2-one/HP-β-CD complex treatment more effectively reduced the weight loss of the potatoes.

#### 3.4.3. Respiration Rate

The respiration rate reflects the intensity of metabolic activity in potato tubers. Potato sprouting significantly increases the respiration rate by breaking dormancy, activating energy metabolism for sprout growth, and enhancing the activity of respiratory enzymes. As storage proceeded, all groups displayed a gradual rise in the respiration rate (Figure 10D). Potatoes in the control group showed the highest respiration rate, reaching 7.25 CO_2_ kg^−1^ h^−1^ on the 70th day due to vigorous sprouting. The 3-decen-2-one treatment reduced potato respiration to a certain extent, but the rate still increased obviously in the later storage stage. In comparison, the 3-decen-2-one/HP-β-CD complex treatment maintained a lower and more stable respiration rate throughout storage.

#### 3.4.4. MDA Content

The MDA content is an important indicator reflecting the degree of cell membrane lipid peroxidation and damage. Potato sprouting accelerates membrane lipid peroxidation, resulting in an increased MDA content and reduced cell membrane integrity. As depicted in Figure 10E, the MDA content in all treatments showed an upward trend during storage. The control group had the highest MDA content, reaching 9.80 nmol g^−1^ on day 70. The 3-decen-2-one treatment effectively inhibited the increase in the MDA content, but the inhibitory effect gradually weakened with prolonged storage. Its MDA level was 8.03 nmol g^−1^ at the end of storage. In contrast, the MDA value for the 3-decen-2-one/HP-β-CD complex-treated group remained significantly lower than those of the other treatments throughout the storage period, with a final level of only 7.67 nmol g^−1^ (*p* < 0.05). These results demonstrated that the inclusion complex could effectively reduce the degree of membrane lipid peroxidation in potatoes.

#### 3.4.5. Vc Content

Vc is an important nutrient component in potato tubers, and its content can reflect the degree of nutrient loss and senescence. During potato storage, Vc is gradually consumed and degraded with the enhancement of respiratory metabolism and sprouting. The variations in the Vc content are illustrated in Figure 10F; the Vc content in all treatments showed a downward trend during storage. The control group had the fastest Vc degradation rate, with only 3.78 mg 100 g^−1^ retained by the 70th day. The 3-decen-2-one treatment slowed down the loss of Vc, but the degradation was still obvious in the later stage. In comparison, the Vc content of the 3-decen-2-one/HP-β-CD inclusion complex treatment was significantly higher than that of the other two groups, reaching 5.43 mg 100 g^−1^ (*p* < 0.05). It was indicated that the inclusion complex could effectively delay Vc degradation in potatoes.

#### 3.4.6. Reducing Sugar Content

Reducing the sugar content is a key index reflecting the processing quality and metabolic characteristics of potato tubers. During sprouting, the content of reducing sugars usually shows a trend of increasing first and then decreasing. In the early stage of sprouting, the activation of amylases promotes starch degradation, producing abundant reducing sugars to support bud germination. As sprouts grow rapidly in the later period, the massive consumption of sugars through intensive respiration exceeds the rate of sugar formation, leading to a decline in the reducing sugar content [46]. As presented in Figure 10G, the reducing sugar content in the control group followed this typical trend: it increased rapidly from 0.25 g 100 g^−1^ to 0.97 g 100 g^−1^ within 28 days, and then decreased to 0.75 g 100 g^−1^ on the 70th day. The 3-decen-2-one treatment effectively suppressed the accumulation of reducing sugars, with the content rising slowly to 0.63 g 100 g^−1^ at the end of storage. In comparison, the 3-decen-2-one/HP-β-CD complex treatment maintained the lowest and most stable reducing sugar content throughout storage, with a final concentration of only 0.53 g 100 g^−1^, which was significantly lower than the control and 3-decen-2-one treatments (*p* < 0.05). These results indicated that the inclusion complex could effectively reduce the accumulation of reducing sugars, thereby better maintaining the processing quality of potato tubers.

## 4. Conclusions

In this study, a 3-decen-2-one/HP-β-CD inclusion complex was prepared by the co-evaporation method. A 1:1 host–guest stoichiometry was confirmed by phase solubility studies. Molecular modeling demonstrated that the inclusion complex was primarily stabilized by van der Waals interactions, and the optimal binding conformation was the alkyl chain of 3-decen-2-one inserted into the cavity from the wider rim of HP-β-CD. The inclusion complex was further characterized by ^1^H NMR, FT-IR, TG, and SEM. Moreover, the aqueous solubility and thermal stability of 3-decen-2-one were significantly improved after the complexation. In addition, the inclusion complex exhibited obvious humidity-responsive release characteristics, which remained stable under low-humidity conditions and slowly released the guest molecule at a high humidity. The inclusion complex also exhibited prominent preservation effects for “Longshu 7” potato tubers during 70 days of storage in corrugated cartons at 15 °C and 95% RH. Specifically, it effectively inhibited potato sprout growth and reduced the sprouting rate, weight loss, respiration rate and MDA accumulation. Meanwhile, it maintained a higher Vc content and a lower reducing sugar content. The complex achieved the sustained release of 3-decen-2-one, and has the potential to overcome the rapid efficacy loss caused by the high volatility of free 3-decen-2-one.

## Figures and Tables

**Table 1:** The thermodynamic parameters of the 3-decen-2-one/HP-β-CD inclusion complex.

	Temperature (°C)				
	20	25	30	35	40
*K* (M^−1^)	596.43	620.03	641.12	656.60	692.08
∆*G* (KJ mol^−1^)	−15.57	−15.92	−16.28	−16.64	−17.00
Δ*H* (KJ mol^−1^)	5.40				
Δ*S* (J mol^−1^ K^−1^)	71.57				

**Table 2:** MM-GBSA decomposed binding free energies of the HP-β-CD/3-decen-2-one complex.

Energy Component	Average	SD (Prop.)	SD	SEM (Prop.)	SEM
ΔVDWAALS	−25.64	0.35	3.02	0.02	0.19
ΔEEL	−2.11	0.26	2.69	0.02	0.17
ΔEGB	9.29	1.83	3.22	0.12	0.2
ΔESURF	−3.57	0.13	0.36	0.01	0.02
ΔGGAS	−27.76	0.44	4.27	0.03	0.27
ΔGSOLV	5.72	1.84	3.15	0.12	0.2
ΔTOTAL	−22.03	1.89	3.42	0.12	0.22

Note: ΔVDWAALS: van der Waals energy; ΔEEL: electrostatic energy; ΔEGB: polar solvation energy; ΔESURF: nonpolar solvation energy; ΔGGAS: gas-phase binding energy; ΔGSOLV: solvation free energy; ΔTOTAL: total binding free energy. SD: standard deviation, SEM: standard error of the mean; SD (Prop.) and SEM(Prop.) represent the standard deviation and standard error of the mean of production trajectory snapshots, respectively. All energy units: kcal mol^−1^.

**Table 3:** Chemical shift values (*δ*/ppm) and variation values (Δ*δ*/ppm) of HP-β-CD before and after proton formation of inclusion complex.

Protons in HP-β-CD Cavity	Chemical Shift Value Before Inclusion, *δ_free_*/ppm	Chemical Shift Value After Inclusion, *δ*_complex_/ppm	Variation Values, Δ*δ*/ppm
H-3	3.887	3.890	+0.003
H-5	3.592	3.603	+0.011

## Data Availability

The original contributions presented in this study are included in the article. Further inquiries can be directed to the corresponding author.
